# Improving 3C-SiC Quality Through Wafer-Bonded Switchback Epitaxy

**DOI:** 10.3390/ma19091896

**Published:** 2026-05-05

**Authors:** Gerard Colston, Kushani H. Perera, Arne Renz, Peter Gammon, Marina Antoniou, Philip A. Mawby, Vishal A. Shah

**Affiliations:** School of Engineering, The University of Warwick, Coventry CV4 7AL, UK; kushani-hapuhinna.perera@warwick.ac.uk (K.H.P.); arne.renz@warwick.ac.uk (A.R.); p.m.gammon@warwick.ac.uk (P.G.); marina.antoniou@warwick.ac.uk (M.A.); p.a.mawby@warwick.ac.uk (P.A.M.); vishal.shah@warwick.ac.uk (V.A.S.)

**Keywords:** 3C-SiC, power electronics, CMP, wafer bonding, epitaxy

## Abstract

The crystallinity of cubic silicon carbide (3C-SiC) epilayers is improved through the use of a novel wafer bonding and regrowth technique resulting in a reduction in planar defects. The process involves the epitaxial growth of a 3–6 µm thick 3C-SiC seed on silicon (Si), which is polished and bonded to a new handle wafer before the original substrate and defective interface region of the 3C-SiC epilayer are removed. Further epitaxial growth on this Bonded Switchback template results in higher quality 3C-SiC epilayers through the reduction in crystal mosaicity, stacking fault defects, and elimination of interface voids. The process could be applied to 3C-SiC grown on both on- and off-axis substrates, and the form of the new handle has no impact on the growth process, enabling this technology to be applied to sapphire or hexagonal 4H-SiC substrates. The use of such substrates would overcome the thermal budget limitations of Si substrates for 3C-SiC heteroepitaxy and ion implantation. Bonded Switchback can improve material quality for applications in power electronics, as well as see the heterogeneous integration of 3C-SiC into other device structures, potentially leading to a new range of hybrid 3C-SiC/Si devices without the high density of defects observed at the interface between these two materials.

## 1. Introduction

Silicon carbide (SiC) is a wide bandgap compound semiconductor that possesses properties that make it ideal for a range of applications, such as those in harsh environments or high voltage power electronics. SiC exists in a number of different crystal structures known as polytypes, and the hexagonal polytype 4H-SiC is the dominant form in the industry [1,2]. Although 4H-SiC is already commercially available as 200 mm diameter substrates, the cost of these wafers and subsequent epitaxy and device fabrication processes are still extremely high, which limits its uptake into mass markets.

SiC can also exist in a cubic (zinc blende) crystal structure, 3C-SiC, which offers several advantages over 4H-SiC and other polytypes. 3C-SiC is metastable, but it can stabilize at lower temperatures and combining this with its cubic (diamond) lattice structure, it can be grown on silicon (Si) substrates, dramatically reducing the starting wafer cost and increasing the substrate quality and variability of specifications, such as crystal orientation, doping, resistivity, offering more exotic growth platforms, such as a silicon on-insulator (SOI) [3,4]. 3C-SiC could offer other advantages over 4H-SiC within power electronics in the 600–1200 V range due to its lower built in voltage [5] and larger 3C-SiC/SiO_2_ barrier, which could increase MOS-based device reliability [6,7].

The heteroepitaxial growth of 3C-SiC is unfortunately made difficult due to the extremely high lattice mismatch (19.7%) between 3C-SiC and Si as well as the high thermal mismatch between the layers, which can lead to wafer bow [8]. 3C-SiC epilayers grown on Si substrates suffer from very high density of misfit dislocations at the interface and planar defects, such as stacking faults, which propagate up throughout the epilayer along the $\left\{111\right\}$ crystal planes. Many of these defects are electrically active, which results in unacceptable leakage currents in rectifying devices, making this material platform unsuitable for applications in power electronics [9]. Another source of defects in 3C-SiC are the boundaries between anti-phase domains (APDs), which are commonly found when growing 3C-SiC on on-axis substrates. Growing 3C-SiC on a substrate with an offcut (typically 4° towards the [110] direction) can eliminate these. Stacking faults will self-annihilate, so increasing epilayer thickness can reduce the surface density of these defects; however, growing thicker 3C-SiC epilayers can increase wafer bow, and a high network of these defects will always exist near the 3C-SiC/Si interface [10]. An upper growth temperature limit is imposed on 3C-SiC epitaxy due to the presence of the underlying Si substrate, which affects the maximum growth rate and ultimate thickness possible with this system. This temperature limitation also has severe impacts on the use of ion-implantation in 3C-SiC, as activation temperatures below 1400 °C are simply not effective at activating dopant carriers [11].

More novel techniques have been explored to reduce the defect density in 3C-SiC, such as through the use of patterned or compliant substrates [12,13,14], through the production of thick 3C-SiC substrates for subsequent homoepitaxial growth [15], and growth on porous Si to reduce lattice mismatch [16]. Another technique of interest is switchback epitaxy, which involves the growth of a 300 µm epilayer of 3C-SiC followed by the removal of the initial substrate by wet chemical etching. The now free-standing epilayer is flipped and thinned by approximately 100 µm, thus removing the region of the 3C-SiC with high defect density. Subsequent epitaxy on this 3C-SiC surface was found to promote further annihilation of defects [17].

This study draws inspiration from the previously reported switchback technology but integrates wafer bonding processes to eliminate the need to grow an extremely thick free-standing 3C-SiC layer. We refer to the process developed within this study as Bonded Switchback, and the impact of this technique on the crystal quality and defect densities within 3C-SiC is investigated, with the aim to develop a higher quality 3C-SiC/Si interface and lower defect 3C-SiC epilayer without relying on extremely thick epitaxial growth.

## 2. Materials and Methods

**Figure 1:**
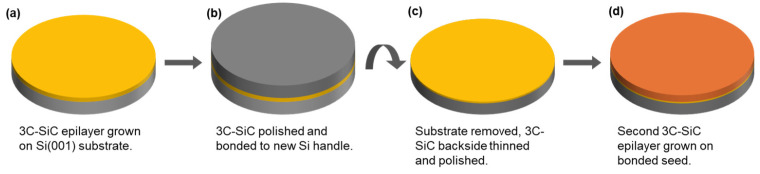
Process flow for the production of Bonded Switchback 3C-SiC epi wafers including (**a**) initial 3C-SiC seed epitaxy, (**b**) CMP and wafer bond of new handle, (**c**) etching of original Si(001) substrate and polishing of 3C-SiC seed epilayer and (**d**) epitaxial growth of second 3C-SiC epilayer, here denoted by darker color.

3C-SiC was grown by reduced pressure chemical vapor deposition within an LPE ACiS M8 RP-CVD at a growth temperature of 1325 °C using trichlorosilane (TCS) and ethylene (C_2_H_4_) as the Si and C precursors, respectively, at a C/Si ratio of 1.4 with a H_2_ carrier gas [14]. A carbonization process was carried out prior to growth at a temperature range of 900–1140 °C to provide a seed layer for thick film epitaxy. The Bonded Switchback process was carried out in several steps. First, a 3–6 µm 3C-SiC epilayer was grown on a 100 mm diameter Si(001) substrate nominally on-axis towards the [110] direction, as shown in Figure 1a. These seeds were found to have a concave wafer bow in the range of 100–150 µm. The epilayer surface roughness was reduced by chemical mechanical polishing (CMP), subcontracted to NovaSiC(Le Bourget-du-Lac, France), to reduce the surface roughness to a level suitable for bonding, typically <500 pm. The surface of the 3C-SiC seed layer was then bonded to a new Si(001) handle wafer through covalent bonding on an EVG ComBond high-vacuum wafer bonding system, as shown in Figure 1b. The process involves a dry-etch step to remove the native oxide of the 3C-SiC and new handle wafer, leaving dangling bonds on each surface and operates under high vacuum (<1 × 10^−7^ mBar) to prevent any native oxide formation. Covalent bonding then occurs under a moderate pressure (typically in the range of a few kN) at room temperature, which minimizes any further wafer bow induced by thermal expansion mismatch. The original substrate was mechanically removed to leave a new 3C-SiC/Si(001) wafer, as shown in Figure 1c, and it was ready for further epitaxy.

**Figure 2:**
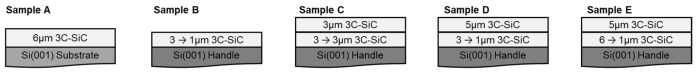
Cross-sectional schematics of the samples investigated.

Several different wafers were produced using this method, as shown in Figure 2. For sample C, the new surface of the 3C-SiC seed layer was only thinned by ~200 nm using CMP to produce a smooth surface for epitaxy. For samples B, D, and E, the CMP process was continued to reduce the thickness of the 3C-SiC seed layer to approximately 1 µm, removing the highly defective first 2–5 µm of growth from the original epilayers. For samples C, D, and E, further epitaxial growth was carried out to bring the total 3C-SiC thickness to 6 µm, and a control growth of 6 µm on a standard Si(001) substrate was included as a reference (sample A). Following epitaxy, samples C-E exhibited approximately 100 µm of convex wafer bow.

The crystal quality of the 3C-SiC samples was assessed by high-resolution X-ray diffraction (HR-XRD) using a Panalytical X’Pert Pro HR-XRD diffractometer. Coupled ω-2θ scans along the symmetric (001) planes confirmed monocrystallinity of the 3C-SiC epilayers, while scanning in the ω axis over the 3C-SiC(002) Bragg peak gives a quantitative measure for the defectiveness and mosaicity of the epilayer. Both cross-sectional (X-TEM) and plan-view (PV-TEM) transmission electron microscopy using a JEOL 2100 TEM were performed to directly observe highly localized areas of the 3C-SiC/Si structures, allowing the density of defects within the 3C-SiC to be estimated. X-TEM and PV-TEM cross-sections were prepared using a focused ion beam scanning electron microscope (FIB-SEM) using a TESCAN Amber FIB-SEM. The surface morphology of 3C-SiC was measured by atomic force microscopy (AFM) using a Bruker Icon operating in Scan Asyst mode.

## 3. Results

**Figure 3:**
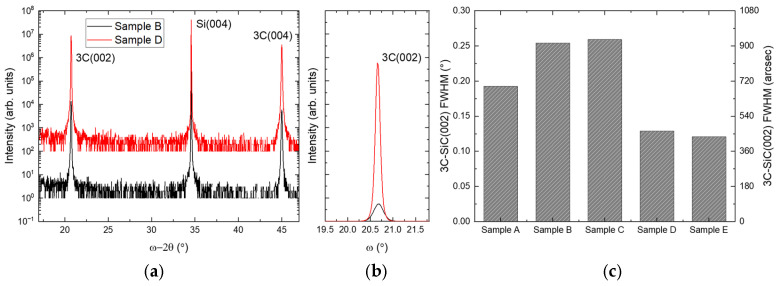
HR-XRD coupled scans (**a**) and ω-rocking curves (**b**) of samples B and D. 3C-SiC(002) FWHM measured by ω-rocking curves (**c**).

The epitaxial growth of 6 µm of 3C-SiC on Si(001) (sample A) produces a single-crystal epilayer with a 3C-SiC(002) Bragg peak of 0.19°, indicating low mosaicity of the crystal, a shown in Figure 3.

**Figure 4:**
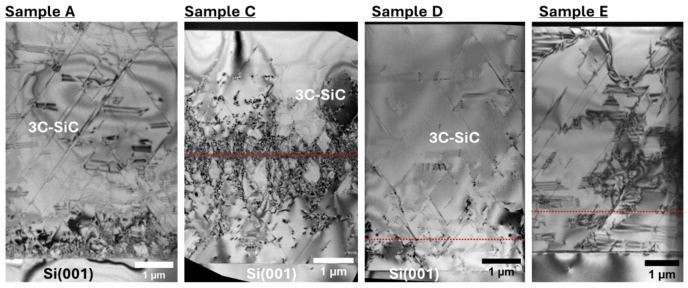
X-TEM micrographs obtained in bright field (004) diffraction condition. The approximate position of the interface between the 3C-SiC seed layer and second epitaxial layer is denoted by a red dotted line.

The density of stacking faults and other planar defects is estimated to be ~5 × 10^4^ cm^−1^ by profiling just below the 3C-SiC surface in X-TEM images; however, the density of planar defects increases dramatically towards the 3C-SiC/Si interface, as shown in Figure 4. The Bonded Switchback process is found to have a varying effect on the quality of the 3C-SiC epilayers. For sample C, no improvement in crystallinity by XRD is observed. The stacking fault density is still high; however, the highest concentration of defects is now at a depth of 3 µm, where the original interface defects were located in the starting 3 µm epilayer from step (a) in Figure 2. An equivalent defect density is observed to penetrate both up and down from the 3 µm in sample B, indicating that flipping the orientation of the initial 3C-SiC is not sufficient to impact the density of planar defects in the layer, as shown in Figure 4.

**Figure 5:**
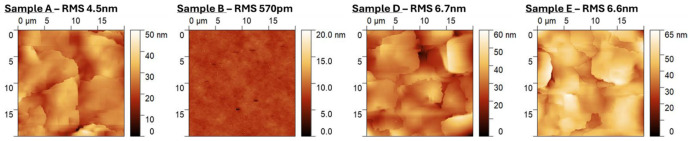
AFM scans of the surface of as-grown, polished, and regrown Bonded Switchback samples.

Samples etched down to 1 µm exhibit a more pronounced effect on the crystal quality. The RMS surface roughness of sample B was measured as 570 pm, confirming that the CMP process was able to smooth the thinned 3C-SiC layer to a suitable level for epitaxial growth, as shown in Figure 5. Growing on this layer shows a reduction in the 3C-SiC(002) FWHM, and a clear improvement compared to the reference sample A is observed for samples D and E, as shown in Figure 3c. The stacking fault density in sample A decreases with increasing epilayer thickness from the Si interface, as expected. The defect density in samples C and D/E peaks at the interface between the two 3C-SiC epilayers at a thickness of 3 µm and 1 µm, respectively. Lines have been added to the images within Figure 4 to indicate the presence of the 3C-SiC/3C-SiC interfaces, which correspond to the highest concentration of planar defects.

The surface morphology of all epitaxial samples indicates the presence of APDs, which is typical of growth upon on-axis substrates, as shown in Figure 5. The presence of these defects persists with Bonded Switchback samples D and E. The RMS roughness of the CMP-only sample B is 570 pm and appears mostly uniform with some evidence of pits in the surface, likely a byproduct of localized poor crystallinity in the original 3C-SiC seed epilayer. The roughness of samples D and E is slightly higher than the reference sample A, suggesting that further optimization of the CMP process is necessary; however, generally, an RMS of this order is considered good for 3C-SiC epilayers.

**Figure 6:**
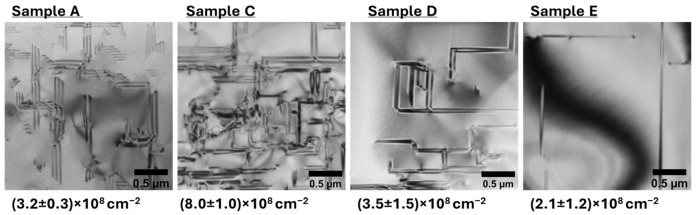
PV-TEM micrographs highlighting defects at the surface of 3C-SiC epilayers; field of view 2.5 µm × 2.5 µm.

PV-TEM lamellae were obtained from the center of epi wafer samples to quantify the area defect density at the surface of the 3C-SiC epilayers. Planar defects are shown as dark lines in the PV-TEM micrographs, and the defect density and uncertainty were estimated from images taken at three separate 2.5 µm × 2.5 µm areas across each extraction, as shown in Figure 6. Sample C exhibits a higher defect density than the control sample A; however, this value reduces as the initial buffer layer thickness is increased and epilayer thinning is implemented, such as in samples D and E.

**Figure 7:**
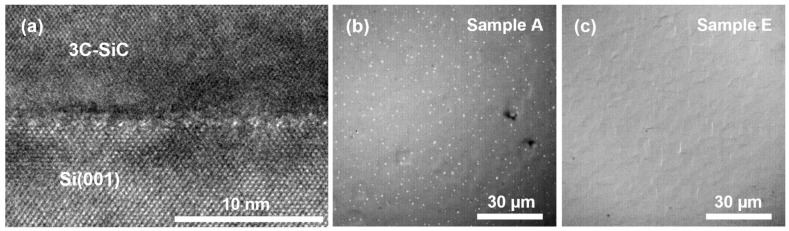
(**a**) X-TEM micrograph of the bonded interface between the polished 3C-SiC and new Si handle wafer of sample C. Differential interference contrast (DIC) optical images of the surface of sample A (**b**), showing the presence of interfacial voids, and sample E (**c**), where no voids are visible.

The state of the bonded interface between the Bonded Switchback 3C-SiC and the new Si handle is observed through lattice-resolved X-TEM imaging, as shown in Figure 7a. A sharp interface between these layers is observed, with both layers retaining crystallinity on both sides. The impact of the bonded interface on the electrical and thermal properties has yet to be fully assessed; however, initial electrical characterization has confirmed that the interface is electrically conductive. Another advantage of this process is the elimination of Si voids typically observed at the 3C-SiC/Si interface, which is confirmed by optical microscopy of the sample, as shown in Figure 7b,c.

**Figure 8:**
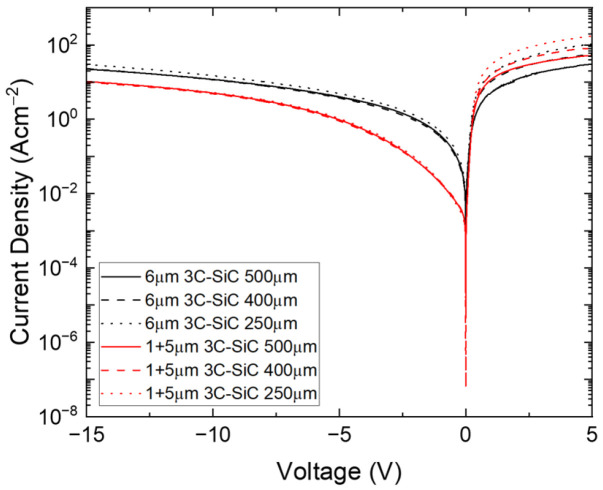
Room temperature I-V characteristics of 3C-SiC Schottky diodes of varying radii (denoted in the legend) fabricated from samples A and E.

Schottky diodes were fabricated using sputtered Pt dots of varying diameters on both samples A and E to compare electrical properties, and a surface NiCr Ohmic contact was used as the drain. The reverse bias characteristics of these devices indicate a reduction in leakage current by 3–5X in the bonded sample, as shown in Figure 8. While other parameters, such as Schottky barrier height, will have an impact on this leakage current, if stacking faults are believed to be a significant contributor of this leakage, then this agrees with the material characterization results of these materials, with sample E exhibiting a lower planar defect density.

## 4. Discussion

A summary of all samples investigated within this report and key quantitative parameters are given in Table 1.

Bonded Switchback is presented as a viable and wafer-scale technique to reduce defect densities within 3C-SiC, relying only on processes standard to the SiC and Si industries. The surface morphology of as-grown 3C-SiC is likely too rough to enable efficient bonding; however, CMP processing enables roughnesses down to ~500 pm RMS, which is demonstrated as sufficient for an abrupt, covalently bonded interface. While epitaxial growth on this polished surface appears to be unaffected, the CMP process itself can introduce contaminants and damage into the 3C-SiC crystal, and further investigation into this is required. The bonded interface is shown to be strong enough to withstand a subsequent epitaxial growth process at 1325 °C. Bonding the 3C-SiC surface and simply removing the original Si substrate generally shows a negative effect on the quality of further epitaxial 3C-SiC (sample C). The controlled removal of the highly defective region of seed 3C-SiC by CMP is crucial and gives a higher quality starting surface for epitaxial growth, and it is shown to reduce mosaicity and overall stacking fault density. This effect is enhanced with the use of a thicker 3C-SiC seed layer, here demonstrated up to 6 µm (sample E). The process is suitable for full wafer-scale production; however, wafer bow must be controlled to levels within the limitations of CMP and wafer bonding techniques, which can be a concern when growing very thick 3C-SiC epilayers.

The process here was demonstrated with on-axis 3C-SiC seed layers only, which tend to exhibit tensile stress and subsequent concave wafer bow. Growth on other crystal orientations, such as (111), is known to suffer from significantly more wafer bow [18], and growth on off-axis (001) wafers can result in “saddle shaped” warp [19], factors that could make it more difficult to apply this technique to 3C-SiC grown on such substrates. The electrical properties of the 3C-SiC grown using the Bonded Switchback process are shown to be improved, which is consistent with the improvement in crystal quality identified through material characterization. The removal of the highly defective region of 3C-SiC could make these epilayers suitable for applications in low-cost power electronics and other device applications.

Another key advantage of the Bonded Switchback technique is that the second epitaxial growth process does not rely on the orientation or even crystallinity of the new handle, and alternatives to Si could enable the fabrication of other device structures through the use of insulating substrates, such as sapphire, or polycrystalline 4H-SiC. Both of these options would also provide improvements to the epitaxial growth of the 3C-SiC, as the upper growth temperature would no longer be limited by the melting point of Si (~1410 °C), and both epitaxial growth and ion implantation annealing of 3C-SiC could be carried out at higher temperatures, which would improve epitaxial growth rates, crystal quality, and dopant activation rates. Further research into the application of this bonding technique to non-silicon substrates is an area of further investigation.

## Figures and Tables

**Table 1:** Sample fabrication and characterization parameter summary.

Sample	Initial 3C Seed (µm)	Final 3C Seed (µm)	2nd Epitaxial Step (µm)	3C(002) FWHM (°)	PV-TEM SF Density (cm^−2^)	RMS (nm)
A	NA	NA	6	0.192	3.2 ± 0.3	4.5
B	3	1	NA	0.254		0.57
C	3	3	3	0.259	8.0 ± 1.0	2.2
D	3	1	5	0.129	3.5 ± 1.5	6.7
E	6	1	5	0.121	2.1 ± 1.2	6.6

## Data Availability

The original contributions presented in this study are included in the article. Further inquiries can be directed to the corresponding author.
